# Crystal structure of methyl 1-methyl-3,5-diphenyl-7-tosyl-3,6,7,11b-tetra­hydro­pyrazolo­[4′,3′:5,6]pyrano[3,4-*c*]quinoline-5a(5*H*)-carboxyl­ate

**DOI:** 10.1107/S160053681402515X

**Published:** 2014-11-29

**Authors:** Eswar Kumar Nadendla, G. Jagadeesan, D. Kannan, Mannickam Bakthadoss, K. Gunasekaran

**Affiliations:** a Centre of Advanced Study in Crystallography and Biophysics, University of Madras, Guindy Campus, Chennai 600 025, India; b Department of Physics, Presidency College, Chennai 600 005, India; c Department of Organic Chemistry, University of Madras, Guindy Campus, Chennai 600 025, India

**Keywords:** crystal structure, sulfonamide, C—H⋯π inter­actions, Thrope–Ingold effect

## Abstract

In the title compound, C_35_H_31_N_3_O_5_S, the piperidine ring adopts an envelope conformation, with the methine C atom as the flap, and the pyran ring adopts a sofa conformation. The mean planes of these two rings are almost normal to one another, making a dihedral angle of 85.96 (5)°. The two phenyl rings, one attached to the pyrazole ring and the other to the pyran ring, are inclined to one another by 65.41 (11)°. They are inclined to the mean planes of the rings to which they are attached by 12.59 (11) and 70.09 (9)°, respectively. There is an intra­molecular C—H⋯π inter­action involving the tosyl­ate methyl group and the phenyl ring attached to the pyrazole ring. In the crystal, mol­ecules are linked by C—H⋯π inter­actions, forming ribbons parallel to (10-2). The ribbons are linked by slipped parallel π–π inter­actions involving inversion-related pyrazole rings [inter-centroid distance = 3.672 (2) Å], forming slabs parallel to (001). A preliminary report of this structure has been published [Bakthadoss *et al.* (2014 ▶). *Eur. J. Org. Chem.* pp. 1505–1513].

## Related literature   

For biological activity of sulfonamide compounds, see: Genç *et al.* (2008 ▶); Özbek *et al.* (2007 ▶); Briganti *et al.* (1997 ▶); Borne *et al.* (1974 ▶); De Clercq (2001 ▶). For details of the Thrope–Ingold effect, see: Bassindale (1984 ▶). For a preliminary report of this structure, see: Bakthadoss *et al.* (2014 ▶).
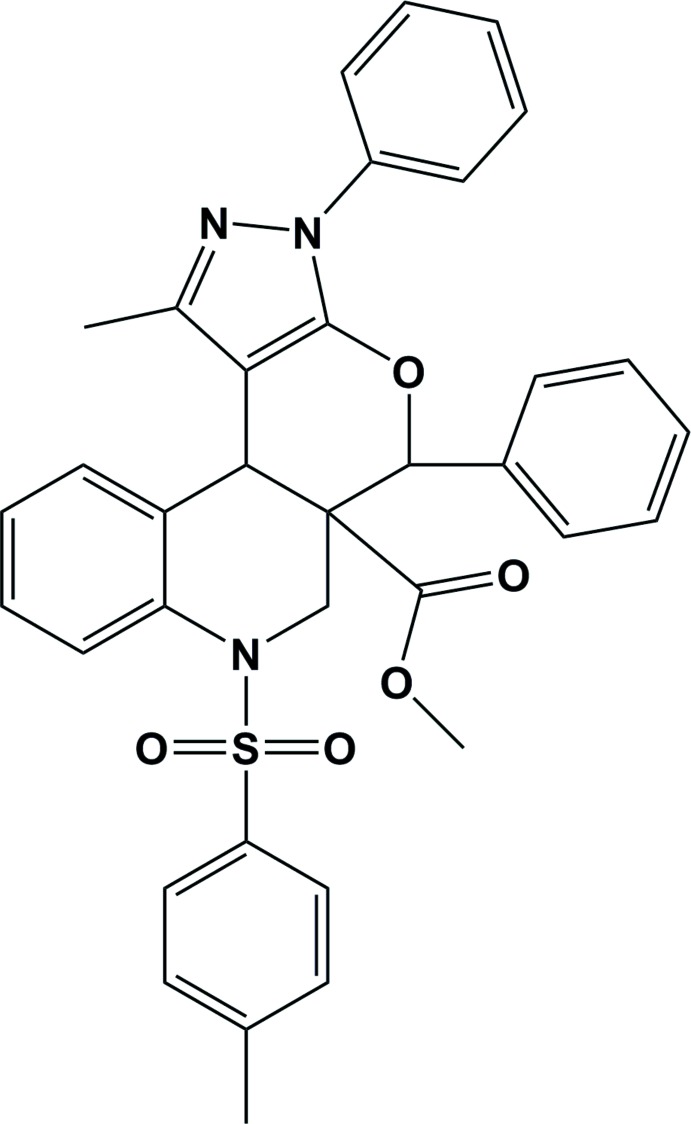


## Experimental   

### Crystal data  

C_35_H_31_N_3_O_5_S
*M* *_r_* = 605.69
Triclinic, 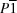

*a* = 10.781 (5) Å
*b* = 11.682 (5) Å
*c* = 14.560 (5) Å
α = 112.708 (5)°
β = 91.908 (5)°
γ = 113.180 (5)°
*V* = 1517.5 (11) Å^3^
*Z* = 2
Mo *K*α radiation
μ = 0.16 mm^−1^
*T* = 293 K
0.25 × 0.20 × 0.20 mm

### Data collection  

Bruker Kappa APEXII CCD diffractometer
Absorption correction: multi-scan (*SADABS*; Bruker, 2004 ▶) *T*_min_ = 0.979, *T*_max_ = 0.983
23138 measured reflections
6308 independent reflections
5070 reflections with *I* > 2σ(*I*)
*R*_int_ = 0.028

### Refinement  

*R*[*F* ^2^ > 2σ(*F* ^2^)] = 0.043
*wR*(*F* ^2^) = 0.118
*S* = 1.04
6308 reflections
406 parameters
H atoms treated by a mixture of independent and constrained refinement
Δρ_max_ = 0.29 e Å^−3^
Δρ_min_ = −0.46 e Å^−3^

### 

Data collection: *APEX2* (Bruker, 2004 ▶); cell refinement: *APEX2* and *SAINT* (Bruker, 2004 ▶); data reduction: *SAINT* and *XPREP* (Bruker, 2004 ▶); program(s) used to solve structure: *SHELXS97* (Sheldrick, 2008 ▶); program(s) used to refine structure: *SHELXL97* (Sheldrick, 2008 ▶); molecular graphics: *PLATON* (Spek, 2009 ▶) and *Mercury* (Macrae *et al.*, 2008 ▶); software used to prepare material for publication: *PLATON*.

## Supplementary Material

Crystal structure: contains datablock(s) I, global. DOI: 10.1107/S160053681402515X/su5015sup1.cif

Structure factors: contains datablock(s) I. DOI: 10.1107/S160053681402515X/su5015Isup2.hkl

Supporting information file. DOI: 10.1107/S160053681402515X/su5015Isup3.cml

**Fig. 1.**
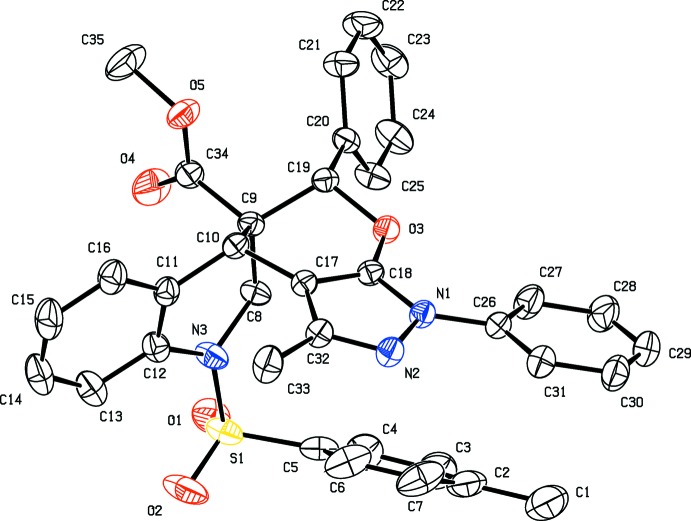
The molecular structure of the title compound, with atom labelling. Displacement ellipsoids are drawn at the 30% probability level (H atoms have been omitted for clarity).

**Fig. 2.**
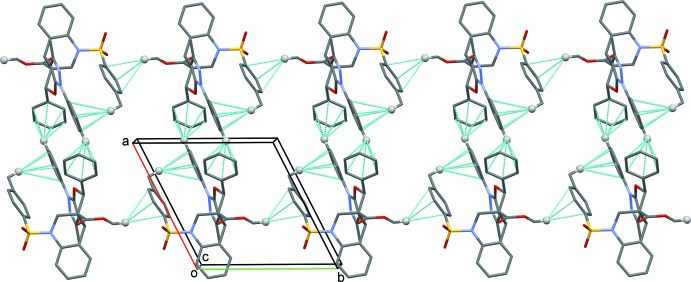
A partial view along the b axis of the crystal packing of the title compound, showing the π-π interaction (red circles represent the centroids of the pyrazole rings; H atoms have been omitted for clarity).

**Fig. 3.**
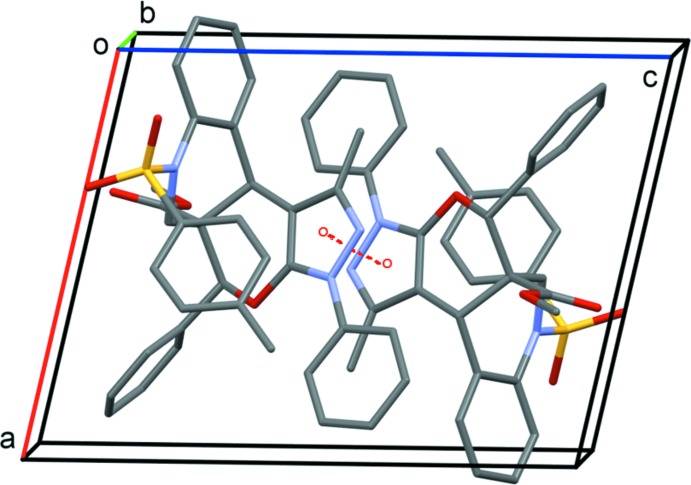
A view along the c axis of the crystal packing of the title compound, showing the C—H···π interactions as dashed lines (H atoms as silver balls; see Table 1 for details; H atoms not involved in these interactions have been omitted for clarity).

. DOI: 10.1107/S160053681402515X/su5015fig1.tif
The mol­ecular structure of the title compound, with atom labelling. Displacement ellipsoids are drawn at the 30% probability level (H atoms have been omitted for clarity).

b . DOI: 10.1107/S160053681402515X/su5015fig2.tif
A partial view along the *b* axis of the crystal packing of the title compound, showing the π-π inter­action (red circles represent the centroids of the pyrazole rings; H atoms have been omitted for clarity).

c . DOI: 10.1107/S160053681402515X/su5015fig3.tif
A view along the *c* axis of the crystal packing of the title compound, showing the C—H⋯π inter­actions as dashed lines (H atoms as silver balls; see Table 1 for details; H atoms not involved in these inter­actions have been omitted for clarity).

CCDC reference: 1034400

Additional supporting information:  crystallographic information; 3D view; checkCIF report

## Figures and Tables

**Table 1:** Hydrogen-bond geometry (, ) *Cg*4, *Cg*6 and *Cg*7 are the centroids of rings C2C7, C20C25 and C26C31, respectively.

*D*H*A*	*D*H	H*A*	*D* *A*	*D*H*A*
C1H1*C* *Cg*7	0.96	2.80	3.682(4)	154
C29H29*Cg*6^i^	0.93	2.94	3.734(3)	144
C35H35*B* *Cg*4^ii^	0.96	2.97	3.853(4)	154

Symmetry codes: (i) 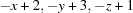; (ii) 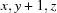.

## supplementary crystallographic information

### S1. Comment

Sulfonamides are widely used as antimicrobial (Genç *et al.*, 2008; Özbek *et al.*, 2007), antifungal (Briganti *et al.*, 1997), anti-inflammatory (Borne *et al.*, 1974) and antiviral agents as well as HIV protease inhibitors (De Clercq *et al.*, 2001). In view of their importance, a series of such compounds were synthesized and the crystal structure of the title compound was briefly reported (Bakthadoss *et al.*, 2014). Herein we report on the full details of the crystal structure of the title compound.

The molecular structure of the title compound is shown in Fig. 1. Atom S1 has a distorted tetrahedral geometry, with the O1—S1—O2 [119.33 (1)°] and N3—S1—C5 [103.46 (1)°] bond angles deviating from the ideal tetrahedral values which is attributed to the Thrope-Ingold effect (Bassindale *et al.*, 1984). The sum of the bond angles around atom N3 (359.22 (2)°) indicates that N3 is sp^2^ hybridized. The pyridine ring (N3/C8—C12) adopts an envelop conformation with atom C10 as the flap; it is displaced by -0.352 (2) Å from the mean formed by the remaining ring atoms. The pyran ring (C9/C10/C17/C18/O3/C19) adopts a sofa conformation [puckering parameters: q2 = 0.434 (2) Å, q3 = 0.358 (2) Å, φ2 = -19.64 (22) °]. The mean planes of these two rings are almost normal to one another with a dihedral angle of 85.96 (5)°. The two different phenyl rings (C26-C31 and C20-C25), attached to the pyrazole (N1/N2/C32/C17/C18) and pyran rings, respectively, are inclined to the mean planes of the rings to which they are attached by 12.59 (11) and 70.09 (9) °, respectively. They are inclined to one another by 65.41 (11) °. There is an intramolecular C-H···π interaction (Table 1) involving the tosylate methyl group, C1, and the phenyl ring (C26-C31) attached to the pyrazole ring.

In the crystal, molecules are linked by C-H···π interactions (Table 1) forming ribbons lying parallel to (102); see Fig. 2. The ribbons are linked via slipped parallel π-π interactions involving inversion related pyrazole rings [Fig. 3; Cg1···Cg1^i^ = 3.672((2) Å; normal distance = 3.565 (1) Å, slippage = 0.882 Å; Cg1 centroid of ring (N1/N2/C18/C17/C32); symmetry code: (i) -x+1, -y+3, -z+1] forming slabs parallel to (001).

### S2. Experimental

A mixture of methyl (2*E*)-2-{[*N*-(2-formylphenyl)(4-methylbenzene) sulfonamido]methyl}-3-phenylprop-2-enoate (0.450 g, 1 mmol) and 3-methyl-1-phenyl-4,5-dihydro-1*H*-pyrazol-5-one (0.174 g, 1 mmol) was placed in a round bottom flask and heated at 453 K for 1 h. After completion of the reaction, as indicated by TLC, the crude product was washed with 5 ml of an ethylacetate/hexane mixture (ratio 1:49) which successfully provided the pure title product as a colourless solid in 96% yield (0.58 g). Diffraction quality crystals were obtained by slow evaporation from an ethyl acetate solution.

### S3. Refinement

Atoms H10, H19 were located in a difference Fourier map and freely refined. The other C-bound H atoms were positioned geometrically and treated as riding atoms, with C—H = 0.93–0.97 Å and with *U*_iso_(H) = 1.5U_eq_(C) for methyl H atoms and = 1.2U_eq_(C) for other H atoms.

### Crystal data

**Table:**

C_35_H_31_N_3_O_5_S	*Z* = 2
*M**_r_* = 605.69	*F*(000) = 636
Triclinic, *P*1	*D*_x_ = 1.326 Mg m^−^^3^
Hall symbol: -P 1	Mo *K*α radiation, λ = 0.71073 Å
*a* = 10.781 (5) Å	Cell parameters from 8834 reflections
*b* = 11.682 (5) Å	θ = 2.1–31.2°
*c* = 14.560 (5) Å	µ = 0.16 mm^−^^1^
α = 112.708 (5)°	*T* = 293 K
β = 91.908 (5)°	Block, colourless
γ = 113.180 (5)°	0.25 × 0.20 × 0.20 mm
*V* = 1517.5 (11) Å^3^	

### Data collection

**Table:**

Bruker Kappa APEXII CCD diffractometer	6308 independent reflections
Radiation source: fine-focus sealed tube	5070 reflections with *I* > 2σ(*I*)
Graphite monochromator	*R*_int_ = 0.028
ω and φ scan	θ_max_ = 26.6°, θ_min_ = 1.6°
Absorption correction: multi-scan (*SADABS*; Bruker, 2004)	*h* = −13→13
*T*_min_ = 0.979, *T*_max_ = 0.983	*k* = −14→14
23138 measured reflections	*l* = −18→18

### Refinement

**Table:**

Refinement on *F*^2^	Secondary atom site location: difference Fourier map
Least-squares matrix: full	Hydrogen site location: inferred from neighbouring sites
*R*[*F*^2^ > 2σ(*F*^2^)] = 0.043	H atoms treated by a mixture of independent and constrained refinement
*wR*(*F*^2^) = 0.118	*w* = 1/[σ^2^(*F*_o_^2^) + (0.0517*P*)^2^ + 0.4727*P*] where *P* = (*F*_o_^2^ + 2*F*_c_^2^)/3
*S* = 1.04	(Δ/σ)_max_ = 0.001
6308 reflections	Δρ_max_ = 0.29 e Å^−^^3^
406 parameters	Δρ_min_ = −0.46 e Å^−^^3^
0 restraints	Extinction correction: *SHELXL97* (Sheldrick, 2008), Fc^*^=kFc[1+0.001xFc^2^λ^3^/sin(2θ)]^-1/4^
Primary atom site location: structure-invariant direct methods	Extinction coefficient: 0.0095 (12)

### Special details

**Table:**

Geometry. All e.s.d.'s (except the e.s.d. in the dihedral angle between two l.s. planes) are estimated using the full covariance matrix. The cell e.s.d.'s are taken into account individually in the estimation of e.s.d.'s in distances, angles and torsion angles; correlations between e.s.d.'s in cell parameters are only used when they are defined by crystal symmetry. An approximate (isotropic) treatment of cell e.s.d.'s is used for estimating e.s.d.'s involving l.s. planes.
Refinement. Refinement of *F*^2^ against ALL reflections. The weighted *R*-factor *wR* and goodness of fit *S* are based on *F*^2^, conventional *R*-factors *R* are based on *F*, with *F* set to zero for negative *F*^2^. The threshold expression of *F*^2^ > σ(*F*^2^) is used only for calculating *R*-factors(gt) *etc*. and is not relevant to the choice of reflections for refinement. *R*-factors based on *F*^2^ are statistically about twice as large as those based on *F*, and *R*- factors based on ALL data will be even larger.

### Fractional atomic coordinates and isotropic or equivalent isotropic displacement parameters (Å^2^)

**Table:**

	*x*	*y*	*z*	*U*_iso_*/*U*_eq_	
H10	0.3360 (16)	1.4361 (17)	0.3161 (12)	0.040 (4)*	
H19	0.5740 (16)	1.5341 (17)	0.3206 (12)	0.039 (4)*	
S1	0.28913 (5)	0.93229 (4)	0.10089 (4)	0.05837 (16)	
O3	0.63192 (11)	1.40325 (11)	0.34307 (8)	0.0427 (3)	
N2	0.44250 (14)	1.28179 (15)	0.50555 (10)	0.0451 (3)	
N1	0.56158 (13)	1.32792 (14)	0.47097 (10)	0.0408 (3)	
O5	0.39810 (15)	1.50993 (13)	0.17850 (9)	0.0585 (3)	
C10	0.33680 (16)	1.34586 (16)	0.28908 (11)	0.0383 (3)	
C18	0.53403 (16)	1.35576 (15)	0.39247 (11)	0.0381 (3)	
C8	0.42521 (16)	1.19731 (15)	0.15787 (12)	0.0428 (4)	
H8A	0.4420	1.1826	0.0901	0.051*	
H8B	0.4977	1.1919	0.1947	0.051*	
C11	0.19167 (16)	1.23858 (17)	0.23342 (12)	0.0428 (4)	
C26	0.68408 (17)	1.33323 (16)	0.51560 (12)	0.0434 (4)	
N3	0.29016 (14)	1.08531 (14)	0.14857 (12)	0.0519 (4)	
C9	0.43402 (16)	1.34228 (15)	0.21259 (11)	0.0374 (3)	
C32	0.34569 (16)	1.28201 (17)	0.44719 (12)	0.0415 (3)	
C19	0.58361 (16)	1.45108 (15)	0.27642 (11)	0.0378 (3)	
C12	0.17045 (17)	1.10761 (18)	0.16377 (12)	0.0463 (4)	
C20	0.69474 (16)	1.48845 (15)	0.21891 (11)	0.0405 (3)	
C17	0.39844 (16)	1.32776 (15)	0.37384 (11)	0.0373 (3)	
C34	0.39288 (18)	1.38461 (17)	0.13379 (12)	0.0471 (4)	
C31	0.6898 (2)	1.31935 (19)	0.60547 (13)	0.0534 (4)	
H31	0.6142	1.3067	0.6361	0.064*	
O4	0.35987 (19)	1.31515 (16)	0.04410 (10)	0.0804 (5)	
O1	0.33189 (16)	0.90828 (14)	0.00616 (10)	0.0696 (4)	
C21	0.7437 (2)	1.61657 (19)	0.21789 (15)	0.0584 (5)	
H21	0.7050	1.6760	0.2501	0.070*	
C33	0.20231 (19)	1.2383 (2)	0.46483 (16)	0.0623 (5)	
H33A	0.1976	1.2124	0.5202	0.093*	
H33B	0.1391	1.1607	0.4041	0.093*	
H33C	0.1779	1.3135	0.4815	0.093*	
C5	0.4190 (2)	0.95070 (16)	0.18862 (14)	0.0535 (4)	
C25	0.75349 (19)	1.40223 (17)	0.17003 (14)	0.0540 (4)	
H25	0.7215	1.3154	0.1694	0.065*	
O2	0.16039 (16)	0.83495 (14)	0.10388 (15)	0.0914 (5)	
C16	0.08020 (19)	1.2695 (2)	0.24679 (15)	0.0574 (5)	
H16	0.0947	1.3581	0.2915	0.069*	
C23	0.9079 (2)	1.5709 (2)	0.12211 (15)	0.0671 (6)	
H23	0.9800	1.5988	0.0903	0.080*	
C13	0.03616 (19)	1.0084 (2)	0.11023 (14)	0.0599 (5)	
H13	0.0207	0.9206	0.0631	0.072*	
C30	0.8089 (2)	1.3243 (2)	0.64975 (16)	0.0653 (5)	
H30	0.8127	1.3152	0.7104	0.078*	
C28	0.9133 (2)	1.3542 (3)	0.5160 (2)	0.0762 (6)	
H28	0.9890	1.3661	0.4855	0.091*	
C14	−0.0727 (2)	1.0410 (3)	0.12734 (16)	0.0709 (6)	
H14	−0.1620	0.9738	0.0926	0.085*	
C24	0.8596 (2)	1.4436 (2)	0.12183 (15)	0.0648 (5)	
H24	0.8983	1.3844	0.0891	0.078*	
C29	0.9205 (2)	1.3424 (2)	0.60559 (18)	0.0706 (6)	
H29	1.0003	1.3465	0.6360	0.085*	
C27	0.7955 (2)	1.3489 (2)	0.46962 (17)	0.0639 (5)	
H27	0.7917	1.3558	0.4082	0.077*	
C4	0.5298 (2)	0.9284 (2)	0.15784 (17)	0.0696 (6)	
H4	0.5400	0.9094	0.0911	0.084*	
C2	0.6146 (3)	0.9648 (2)	0.32640 (18)	0.0749 (6)	
C6	0.4065 (3)	0.9812 (2)	0.28845 (17)	0.0773 (7)	
H6	0.3319	0.9970	0.3099	0.093*	
C15	−0.0524 (2)	1.1702 (3)	0.19444 (18)	0.0717 (6)	
H15	−0.1270	1.1911	0.2048	0.086*	
C22	0.8497 (2)	1.6572 (2)	0.16937 (18)	0.0731 (6)	
H22	0.8814	1.7433	0.1689	0.088*	
C1	0.7210 (4)	0.9729 (3)	0.4012 (2)	0.1103 (10)	
H1A	0.7904	0.9537	0.3676	0.165*	
H1B	0.6772	0.9062	0.4272	0.165*	
H1C	0.7633	1.0639	0.4566	0.165*	
C3	0.6255 (3)	0.9343 (3)	0.2266 (2)	0.0811 (7)	
H3	0.6994	0.9173	0.2051	0.097*	
C7	0.5029 (3)	0.9882 (3)	0.35505 (18)	0.0877 (8)	
H7	0.4935	1.0093	0.4221	0.105*	
C35	0.3590 (3)	1.5611 (3)	0.1114 (2)	0.0913 (8)	
H35B	0.3666	1.6518	0.1513	0.137*	
H35A	0.2653	1.4999	0.0741	0.137*	
H35C	0.4193	1.5652	0.0645	0.137*	

### Atomic displacement parameters (Å^2^)

**Table:**

	*U*^11^	*U*^22^	*U*^33^	*U*^12^	*U*^13^	*U*^23^
S1	0.0555 (3)	0.0336 (2)	0.0674 (3)	0.00953 (19)	0.0169 (2)	0.0141 (2)
O3	0.0370 (6)	0.0510 (6)	0.0478 (6)	0.0183 (5)	0.0128 (5)	0.0297 (5)
N2	0.0434 (8)	0.0582 (8)	0.0469 (7)	0.0262 (7)	0.0174 (6)	0.0310 (7)
N1	0.0384 (7)	0.0492 (7)	0.0440 (7)	0.0221 (6)	0.0125 (6)	0.0260 (6)
O5	0.0812 (9)	0.0553 (7)	0.0515 (7)	0.0362 (7)	0.0097 (6)	0.0295 (6)
C10	0.0407 (8)	0.0399 (8)	0.0391 (8)	0.0208 (7)	0.0108 (6)	0.0186 (6)
C18	0.0402 (8)	0.0386 (7)	0.0391 (8)	0.0183 (7)	0.0116 (6)	0.0192 (6)
C8	0.0405 (9)	0.0353 (8)	0.0470 (9)	0.0120 (7)	0.0154 (7)	0.0169 (7)
C11	0.0390 (9)	0.0544 (9)	0.0410 (8)	0.0204 (7)	0.0103 (7)	0.0266 (7)
C26	0.0420 (9)	0.0413 (8)	0.0499 (9)	0.0203 (7)	0.0061 (7)	0.0211 (7)
N3	0.0405 (8)	0.0370 (7)	0.0665 (9)	0.0105 (6)	0.0173 (7)	0.0174 (7)
C9	0.0388 (8)	0.0355 (7)	0.0370 (7)	0.0141 (6)	0.0097 (6)	0.0171 (6)
C32	0.0421 (9)	0.0495 (9)	0.0411 (8)	0.0243 (7)	0.0150 (7)	0.0227 (7)
C19	0.0422 (9)	0.0343 (7)	0.0374 (7)	0.0157 (7)	0.0091 (6)	0.0171 (6)
C12	0.0392 (9)	0.0543 (9)	0.0416 (8)	0.0146 (7)	0.0103 (7)	0.0231 (7)
C20	0.0402 (8)	0.0360 (7)	0.0373 (8)	0.0096 (6)	0.0067 (6)	0.0156 (6)
C17	0.0383 (8)	0.0390 (7)	0.0379 (7)	0.0193 (6)	0.0110 (6)	0.0175 (6)
C34	0.0473 (10)	0.0483 (9)	0.0432 (9)	0.0157 (8)	0.0085 (7)	0.0232 (7)
C31	0.0554 (11)	0.0603 (11)	0.0497 (10)	0.0301 (9)	0.0089 (8)	0.0245 (8)
O4	0.1224 (14)	0.0725 (9)	0.0388 (7)	0.0412 (9)	0.0013 (8)	0.0200 (7)
O1	0.0846 (10)	0.0541 (8)	0.0490 (7)	0.0274 (7)	0.0122 (7)	0.0056 (6)
C21	0.0624 (12)	0.0466 (9)	0.0712 (12)	0.0200 (9)	0.0212 (10)	0.0340 (9)
C33	0.0481 (11)	0.0974 (15)	0.0655 (12)	0.0370 (11)	0.0260 (9)	0.0524 (11)
C5	0.0718 (12)	0.0342 (8)	0.0576 (10)	0.0222 (8)	0.0291 (9)	0.0231 (7)
C25	0.0528 (10)	0.0390 (8)	0.0604 (10)	0.0135 (8)	0.0218 (8)	0.0181 (8)
O2	0.0651 (10)	0.0440 (8)	0.1374 (15)	0.0041 (7)	0.0300 (10)	0.0326 (9)
C16	0.0482 (11)	0.0734 (12)	0.0613 (11)	0.0323 (10)	0.0156 (9)	0.0337 (10)
C23	0.0573 (12)	0.0717 (13)	0.0573 (11)	0.0091 (10)	0.0221 (9)	0.0326 (10)
C13	0.0459 (10)	0.0661 (12)	0.0472 (10)	0.0098 (9)	0.0079 (8)	0.0202 (9)
C30	0.0686 (13)	0.0733 (13)	0.0588 (11)	0.0372 (11)	−0.0015 (10)	0.0284 (10)
C28	0.0486 (12)	0.0986 (17)	0.1131 (19)	0.0394 (12)	0.0253 (12)	0.0689 (15)
C14	0.0384 (10)	0.0953 (17)	0.0600 (12)	0.0134 (10)	0.0042 (9)	0.0325 (12)
C24	0.0549 (11)	0.0579 (11)	0.0605 (11)	0.0144 (9)	0.0250 (9)	0.0149 (9)
C29	0.0519 (12)	0.0750 (13)	0.0912 (15)	0.0313 (11)	−0.0010 (11)	0.0400 (12)
C27	0.0512 (11)	0.0893 (14)	0.0827 (14)	0.0388 (11)	0.0244 (10)	0.0591 (12)
C4	0.0756 (14)	0.0833 (14)	0.0634 (12)	0.0378 (12)	0.0333 (11)	0.0405 (11)
C2	0.1020 (18)	0.0482 (11)	0.0754 (14)	0.0260 (11)	0.0109 (13)	0.0356 (10)
C6	0.123 (2)	0.0769 (14)	0.0718 (14)	0.0663 (15)	0.0543 (14)	0.0453 (12)
C15	0.0422 (11)	0.1032 (18)	0.0780 (14)	0.0350 (12)	0.0131 (10)	0.0444 (14)
C22	0.0742 (14)	0.0611 (12)	0.0874 (15)	0.0142 (11)	0.0269 (12)	0.0508 (12)
C1	0.146 (3)	0.0780 (17)	0.106 (2)	0.0447 (18)	−0.0054 (19)	0.0468 (16)
C3	0.0771 (16)	0.0935 (17)	0.0934 (17)	0.0399 (14)	0.0352 (13)	0.0570 (14)
C7	0.155 (3)	0.0794 (15)	0.0605 (13)	0.0726 (18)	0.0419 (15)	0.0387 (12)
C35	0.134 (2)	0.0933 (17)	0.0816 (16)	0.0638 (17)	0.0154 (15)	0.0578 (14)

### Geometric parameters (Å, º)

**Table:**

S1—O2	1.4217 (16)	C33—H33B	0.9600
S1—O1	1.4279 (15)	C33—H33C	0.9600
S1—N3	1.6442 (16)	C5—C4	1.373 (3)
S1—C5	1.752 (2)	C5—C6	1.382 (3)
O3—C18	1.3552 (18)	C25—C24	1.384 (2)
O3—C19	1.4627 (18)	C25—H25	0.9300
N2—C32	1.325 (2)	C16—C15	1.382 (3)
N2—N1	1.3784 (18)	C16—H16	0.9300
N1—C18	1.3567 (19)	C23—C24	1.367 (3)
N1—C26	1.420 (2)	C23—C22	1.369 (3)
O5—C34	1.329 (2)	C23—H23	0.9300
O5—C35	1.453 (2)	C13—C14	1.372 (3)
C10—C11	1.504 (2)	C13—H13	0.9300
C10—C17	1.506 (2)	C30—C29	1.365 (3)
C10—C9	1.555 (2)	C30—H30	0.9300
C10—H10	0.977 (16)	C28—C29	1.367 (3)
C18—C17	1.359 (2)	C28—C27	1.385 (3)
C8—N3	1.483 (2)	C28—H28	0.9300
C8—C9	1.529 (2)	C14—C15	1.367 (3)
C8—H8A	0.9700	C14—H14	0.9300
C8—H8B	0.9700	C24—H24	0.9300
C11—C16	1.384 (2)	C29—H29	0.9300
C11—C12	1.390 (2)	C27—H27	0.9300
C26—C27	1.378 (3)	C4—C3	1.376 (3)
C26—C31	1.382 (2)	C4—H4	0.9300
N3—C12	1.422 (2)	C2—C3	1.377 (3)
C9—C34	1.528 (2)	C2—C7	1.380 (4)
C9—C19	1.567 (2)	C2—C1	1.505 (4)
C32—C17	1.411 (2)	C6—C7	1.354 (4)
C32—C33	1.491 (2)	C6—H6	0.9300
C19—C20	1.505 (2)	C15—H15	0.9300
C19—H19	0.983 (16)	C22—H22	0.9300
C12—C13	1.398 (3)	C1—H1A	0.9600
C20—C25	1.379 (2)	C1—H1B	0.9600
C20—C21	1.384 (2)	C1—H1C	0.9600
C34—O4	1.189 (2)	C3—H3	0.9300
C31—C30	1.387 (3)	C7—H7	0.9300
C31—H31	0.9300	C35—H35B	0.9600
C21—C22	1.384 (3)	C35—H35A	0.9600
C21—H21	0.9300	C35—H35C	0.9600
C33—H33A	0.9600		
			
O2—S1—O1	119.29 (10)	H33A—C33—H33B	109.5
O2—S1—N3	108.19 (9)	C32—C33—H33C	109.5
O1—S1—N3	108.89 (8)	H33A—C33—H33C	109.5
O2—S1—C5	108.09 (11)	H33B—C33—H33C	109.5
O1—S1—C5	107.78 (9)	C4—C5—C6	119.6 (2)
N3—S1—C5	103.47 (8)	C4—C5—S1	120.53 (15)
C18—O3—C19	111.44 (12)	C6—C5—S1	119.75 (17)
C32—N2—N1	105.70 (12)	C20—C25—C24	120.66 (17)
C18—N1—N2	109.14 (12)	C20—C25—H25	119.7
C18—N1—C26	131.38 (13)	C24—C25—H25	119.7
N2—N1—C26	119.42 (12)	C15—C16—C11	120.7 (2)
C34—O5—C35	116.36 (16)	C15—C16—H16	119.6
C11—C10—C17	115.19 (13)	C11—C16—H16	119.6
C11—C10—C9	109.36 (13)	C24—C23—C22	119.68 (18)
C17—C10—C9	106.39 (12)	C24—C23—H23	120.2
C11—C10—H10	107.5 (10)	C22—C23—H23	120.2
C17—C10—H10	109.8 (9)	C14—C13—C12	119.8 (2)
C9—C10—H10	108.4 (9)	C14—C13—H13	120.1
O3—C18—N1	122.47 (14)	C12—C13—H13	120.1
O3—C18—C17	127.94 (14)	C29—C30—C31	120.89 (19)
N1—C18—C17	109.59 (13)	C29—C30—H30	119.6
N3—C8—C9	113.72 (13)	C31—C30—H30	119.6
N3—C8—H8A	108.8	C29—C28—C27	121.4 (2)
C9—C8—H8A	108.8	C29—C28—H28	119.3
N3—C8—H8B	108.8	C27—C28—H28	119.3
C9—C8—H8B	108.8	C15—C14—C13	121.25 (19)
H8A—C8—H8B	107.7	C15—C14—H14	119.4
C16—C11—C12	119.56 (16)	C13—C14—H14	119.4
C16—C11—C10	121.39 (16)	C23—C24—C25	120.4 (2)
C12—C11—C10	118.99 (14)	C23—C24—H24	119.8
C27—C26—C31	119.75 (16)	C25—C24—H24	119.8
C27—C26—N1	121.13 (15)	C30—C29—C28	119.08 (19)
C31—C26—N1	119.11 (15)	C30—C29—H29	120.5
C12—N3—C8	122.18 (14)	C28—C29—H29	120.5
C12—N3—S1	124.35 (11)	C26—C27—C28	119.28 (19)
C8—N3—S1	112.69 (11)	C26—C27—H27	120.4
C34—C9—C8	109.29 (13)	C28—C27—H27	120.4
C34—C9—C10	109.65 (13)	C5—C4—C3	119.4 (2)
C8—C9—C10	110.66 (12)	C5—C4—H4	120.3
C34—C9—C19	109.12 (12)	C3—C4—H4	120.3
C8—C9—C19	111.53 (13)	C3—C2—C7	117.4 (2)
C10—C9—C19	106.54 (12)	C3—C2—C1	121.1 (3)
N2—C32—C17	111.64 (14)	C7—C2—C1	121.4 (2)
N2—C32—C33	119.83 (14)	C7—C6—C5	119.9 (2)
C17—C32—C33	128.53 (15)	C7—C6—H6	120.1
O3—C19—C20	106.54 (12)	C5—C6—H6	120.1
O3—C19—C9	109.95 (11)	C14—C15—C16	119.4 (2)
C20—C19—C9	117.66 (12)	C14—C15—H15	120.3
O3—C19—H19	107.2 (9)	C16—C15—H15	120.3
C20—C19—H19	109.9 (9)	C23—C22—C21	120.24 (18)
C9—C19—H19	105.2 (10)	C23—C22—H22	119.9
C11—C12—C13	119.26 (17)	C21—C22—H22	119.9
C11—C12—N3	116.79 (15)	C2—C1—H1A	109.5
C13—C12—N3	123.90 (17)	C2—C1—H1B	109.5
C25—C20—C21	118.44 (16)	H1A—C1—H1B	109.5
C25—C20—C19	122.73 (14)	C2—C1—H1C	109.5
C21—C20—C19	118.79 (15)	H1A—C1—H1C	109.5
C18—C17—C32	103.92 (13)	H1B—C1—H1C	109.5
C18—C17—C10	121.65 (13)	C4—C3—C2	121.6 (2)
C32—C17—C10	134.42 (14)	C4—C3—H3	119.2
O4—C34—O5	123.95 (16)	C2—C3—H3	119.2
O4—C34—C9	124.90 (16)	C6—C7—C2	122.0 (2)
O5—C34—C9	111.15 (13)	C6—C7—H7	119.0
C26—C31—C30	119.63 (18)	C2—C7—H7	119.0
C26—C31—H31	120.2	O5—C35—H35B	109.5
C30—C31—H31	120.2	O5—C35—H35A	109.5
C20—C21—C22	120.60 (19)	H35B—C35—H35A	109.5
C20—C21—H21	119.7	O5—C35—H35C	109.5
C22—C21—H21	119.7	H35B—C35—H35C	109.5
C32—C33—H33A	109.5	H35A—C35—H35C	109.5
C32—C33—H33B	109.5		
			
C32—N2—N1—C18	0.31 (17)	O3—C18—C17—C10	0.9 (2)
C32—N2—N1—C26	−177.06 (14)	N1—C18—C17—C10	−178.65 (13)
C19—O3—C18—N1	−168.50 (13)	N2—C32—C17—C18	−0.31 (18)
C19—O3—C18—C17	12.0 (2)	C33—C32—C17—C18	179.12 (18)
N2—N1—C18—O3	179.90 (13)	N2—C32—C17—C10	178.67 (16)
C26—N1—C18—O3	−3.2 (3)	C33—C32—C17—C10	−1.9 (3)
N2—N1—C18—C17	−0.52 (17)	C11—C10—C17—C18	142.60 (15)
C26—N1—C18—C17	176.42 (15)	C9—C10—C17—C18	21.25 (19)
C17—C10—C11—C16	107.29 (17)	C11—C10—C17—C32	−36.2 (2)
C9—C10—C11—C16	−132.99 (15)	C9—C10—C17—C32	−157.59 (17)
C17—C10—C11—C12	−75.64 (18)	C35—O5—C34—O4	0.7 (3)
C9—C10—C11—C12	44.08 (18)	C35—O5—C34—C9	−179.09 (17)
C18—N1—C26—C27	−11.1 (3)	C8—C9—C34—O4	−0.3 (2)
N2—N1—C26—C27	165.55 (16)	C10—C9—C34—O4	−121.7 (2)
C18—N1—C26—C31	170.30 (16)	C19—C9—C34—O4	121.9 (2)
N2—N1—C26—C31	−13.0 (2)	C8—C9—C34—O5	179.48 (13)
C9—C8—N3—C12	15.9 (2)	C10—C9—C34—O5	58.02 (17)
C9—C8—N3—S1	−173.73 (11)	C19—C9—C34—O5	−58.32 (18)
O2—S1—N3—C12	−15.17 (18)	C27—C26—C31—C30	1.4 (3)
O1—S1—N3—C12	115.88 (15)	N1—C26—C31—C30	179.97 (16)
C5—S1—N3—C12	−129.68 (15)	C25—C20—C21—C22	−0.4 (3)
O2—S1—N3—C8	174.75 (13)	C19—C20—C21—C22	177.39 (18)
O1—S1—N3—C8	−54.20 (15)	O2—S1—C5—C4	124.05 (17)
C5—S1—N3—C8	60.24 (14)	O1—S1—C5—C4	−6.13 (18)
N3—C8—C9—C34	−92.95 (16)	N3—S1—C5—C4	−121.37 (16)
N3—C8—C9—C10	27.89 (18)	O2—S1—C5—C6	−52.72 (18)
N3—C8—C9—C19	146.30 (14)	O1—S1—C5—C6	177.09 (15)
C11—C10—C9—C34	64.66 (16)	N3—S1—C5—C6	61.85 (16)
C17—C10—C9—C34	−170.33 (12)	C21—C20—C25—C24	0.5 (3)
C11—C10—C9—C8	−55.97 (16)	C19—C20—C25—C24	−177.17 (17)
C17—C10—C9—C8	69.04 (15)	C12—C11—C16—C15	2.4 (3)
C11—C10—C9—C19	−177.37 (12)	C10—C11—C16—C15	179.42 (17)
C17—C10—C9—C19	−52.37 (15)	C11—C12—C13—C14	−0.4 (3)
N1—N2—C32—C17	0.00 (18)	N3—C12—C13—C14	−177.57 (17)
N1—N2—C32—C33	−179.48 (16)	C26—C31—C30—C29	−0.2 (3)
C18—O3—C19—C20	−174.85 (12)	C12—C13—C14—C15	1.5 (3)
C18—O3—C19—C9	−46.32 (16)	C22—C23—C24—C25	−0.8 (3)
C34—C9—C19—O3	−172.63 (12)	C20—C25—C24—C23	0.1 (3)
C8—C9—C19—O3	−51.79 (16)	C31—C30—C29—C28	−0.6 (3)
C10—C9—C19—O3	69.06 (15)	C27—C28—C29—C30	0.3 (4)
C34—C9—C19—C20	−50.48 (18)	C31—C26—C27—C28	−1.7 (3)
C8—C9—C19—C20	70.36 (16)	N1—C26—C27—C28	179.71 (19)
C10—C9—C19—C20	−168.78 (13)	C29—C28—C27—C26	0.9 (4)
C16—C11—C12—C13	−1.5 (2)	C6—C5—C4—C3	1.2 (3)
C10—C11—C12—C13	−178.65 (14)	S1—C5—C4—C3	−175.61 (17)
C16—C11—C12—N3	175.88 (15)	C4—C5—C6—C7	−0.4 (3)
C10—C11—C12—N3	−1.3 (2)	S1—C5—C6—C7	176.45 (18)
C8—N3—C12—C11	−31.4 (2)	C13—C14—C15—C16	−0.7 (3)
S1—N3—C12—C11	159.43 (13)	C11—C16—C15—C14	−1.3 (3)
C8—N3—C12—C13	145.87 (17)	C24—C23—C22—C21	0.9 (3)
S1—N3—C12—C13	−23.3 (2)	C20—C21—C22—C23	−0.3 (3)
O3—C19—C20—C25	48.67 (19)	C5—C4—C3—C2	−1.3 (4)
C9—C19—C20—C25	−75.2 (2)	C7—C2—C3—C4	0.6 (3)
O3—C19—C20—C21	−129.00 (16)	C1—C2—C3—C4	−179.1 (2)
C9—C19—C20—C21	107.12 (17)	C5—C6—C7—C2	−0.4 (4)
O3—C18—C17—C32	−179.96 (15)	C3—C2—C7—C6	0.3 (4)
N1—C18—C17—C32	0.49 (17)	C1—C2—C7—C6	180.0 (2)

### Hydrogen-bond geometry (Å, º)

Cg4, Cg6 and Cg7 are the centroids of rings C2–C7, C20–C25 and C26–C31, respectively.

**Table:**

*D*—H···*A*	*D*—H	H···*A*	*D*···*A*	*D*—H···*A*
C1—H1*C*···*Cg*7	0.96	2.80	3.682 (4)	154
C29—H29···*Cg*6^i^	0.93	2.94	3.734 (3)	144
C35—H35*B*···*Cg*4^ii^	0.96	2.97	3.853 (4)	154

Symmetry codes: (i) −*x*+2, −*y*+3, −*z*+1; (ii) *x*, *y*+1, *z*.
