# Supplementary figures and images for: Crystal structure of methyl 1-methyl-3,5-diphenyl-7-tosyl-3,6,7,11b-tetra­hydro­pyrazolo­[4′,3′:5,6]pyrano[3,4-c]quinoline-5a(5H)-carboxyl­ate
Source: Acta Crystallogr Sect E Struct Rep Online. 2014 Nov 29;70(Pt 12):o1295–6. doi: 10.1107/S160053681402515X (PMC4257454; doi:10.1107/S160053681402515X)

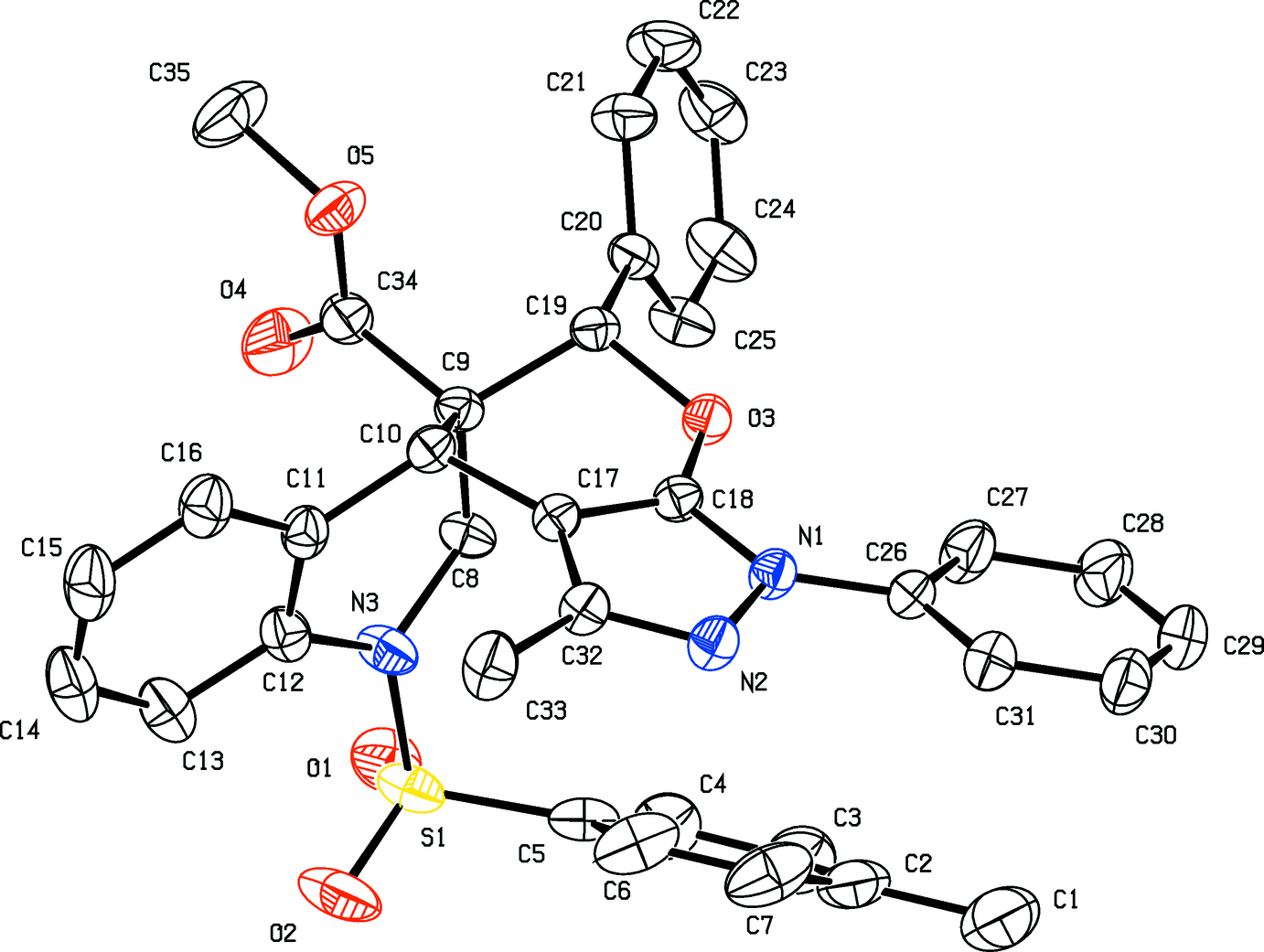

Supplement: Supplementary file 4 [file e-70-o1295-fig1.tif]

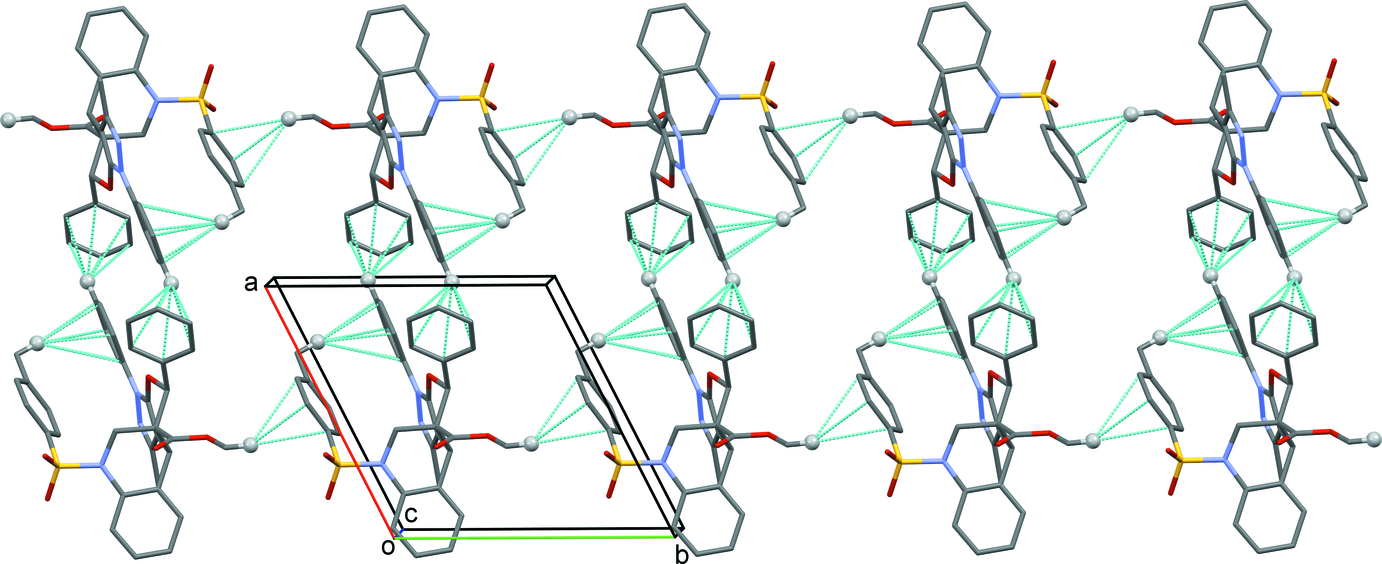

Supplement: Supplementary file 5 [file e-70-o1295-fig2.tif]

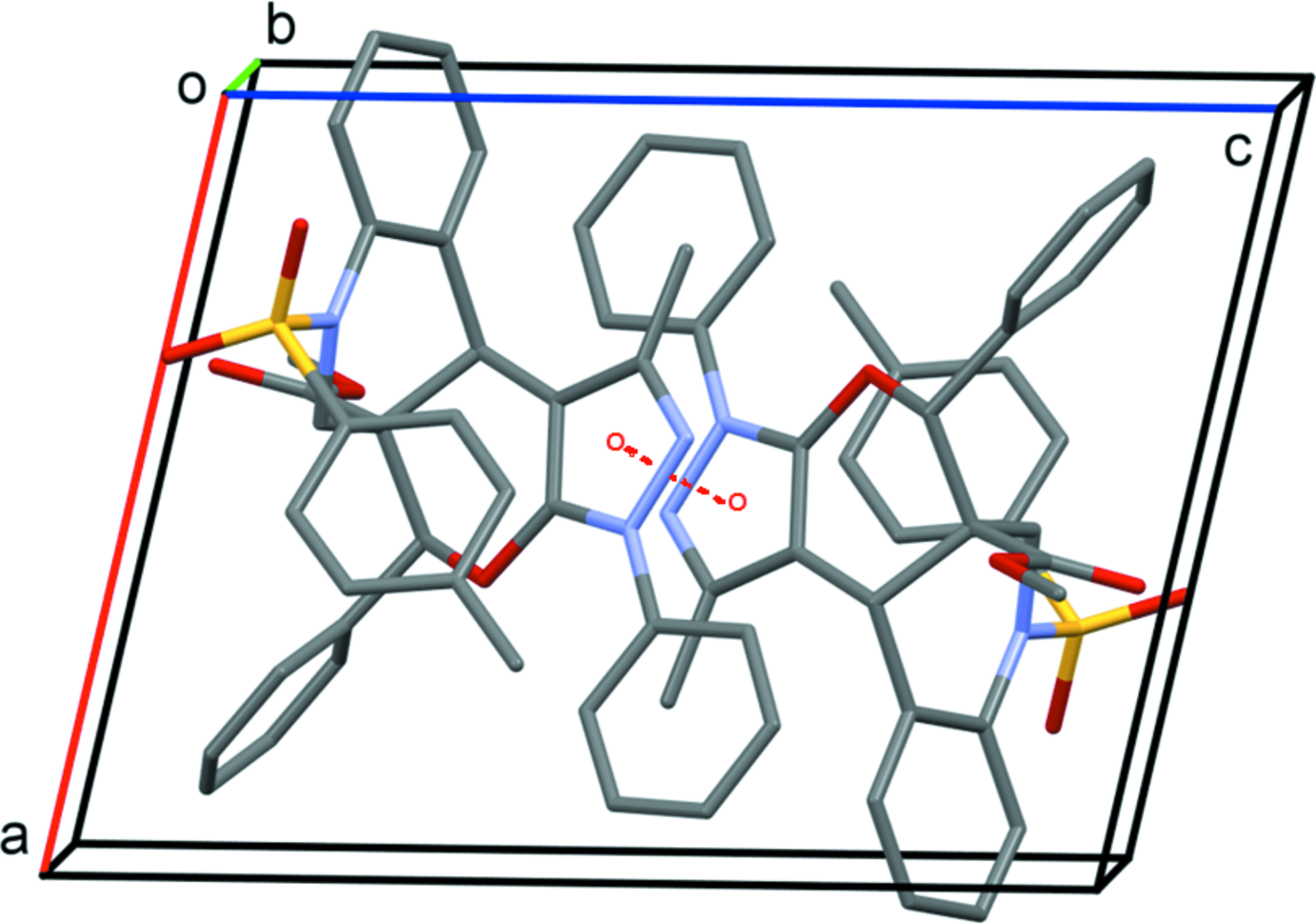

Supplement: Supplementary file 6 [file e-70-o1295-fig3.tif]
